# Prospects for the control of Bolivian hemorrhagic fever.

**DOI:** 10.3201/eid0103.950308

**Published:** 1995

**Authors:** P E Kilgore, C J Peters, J N Mills, P E Rollin, L Armstrong, A S Khan, T G Ksiazek


was a Zaire subtype that differed from the original 1976 strain in four bases (<1%). No differences were seen when the polymerase gene PCR products (~350 bp) from those four patients were sequenced, which indicated that they had been infected with the same virus. Three days later, sequence data from expanded analysis of the entire glycoprotein gene were compared with those of the original 1976 Yambuku isolate (9) and showed that the overall difference between these Ebola viruses was less than 1.6%. Such little change in viruses that caused outbreaks of disease at extreme ends of Zaire separated by a span of nearly 19 years, may indicate that the genomes of Ebola viruses (and filoviruses in general) are unusually stable and have evolved to occupy special niches in the wild.

The capability to rapidly diagnose and characterize filovirus infections is critical to the ability of public health professionals to identify and limit the spread of future outbreaks of filovirus disease. A continued commitment to research and modern disease-surveillance programs is necessary to minimize or preclude filovirus outbreaks similar to that in Kikwit. The possibility of outbreaks is increasingly likely given the continued human incursions into the African forests and the vulnerability of large impoverished populations to rapid transmission of disease as a result of inadequate public health services. With the current outbreak under control, CDC and collaborators have begun their efforts to identify the natural reservoir by sending teams of scientists to collect specimens from the area where the putative index patient worked. Attempts to identify the reservoir after outbreaks in 1976 and 1979 were handicapped by the lack of satisfactory diagnostic tools that are critical to detecting small quantities of the virus. However, now that sensitive enzyme immunoassays and PCR assays have been developed for filoviruses, the chances are much better that, if appropriate materials can be collected in the field, the virus can be detected.

In conclusion, we want to alert physicians and public health agencies who encounter persons that have clinical signs and symptoms of hemorrhagic fever disease to the reemergence of Ebola virus. Recommendations for the management of viral hemorrhagic fevers attributable to filoviruses in the United States were recently published in CDC's *Morbidity and Mortality Weekly Report* (1995;44:475-79).

**Anthony Sanchez, Thomas G. Ksiazek, Pierre E. Rollin, Clarence J. Peters, Stuart T. Nichol, Ali S. Khan, and Brian W. J. Mahy**
National Center for Infectious Diseases
Centers for Disease Control and Prevention
Atlanta, Georgia, USA

### References

1. Le Guenno B, Formenty P, Wyers M, Gounon P, Walker F, Boesch C. Isolation and partial characterisation of a new strain of Ebola virus. Lancet 1995;345:1271-4
2. Centers for Disease Control and Prevention. Outbreak of Ebola viral hemorrhagic fever—Zaire, 1995. MMWR 1995;44:381-2.
3. Centers for Disease Control and Prevention. Update: outbreak of ebola viral hemorrhagic fever—Zaire, 1995. MMWR 1995;44:399.
4. Bowen ETW, Platt GS, Lloyd G, Baskerville A, Harris WJ, Vella EC. Viral haemorrhagic fever in southern Sudan and northern Zaire: preliminary studies on the aetiologic agent. Lancet 1977;1:571-3.
5. Baron RC, McCormick JB, Zubeir OA. Ebola virus disease in southern Sudan: hospital dissemination and intrafamilial spread. Bull WHO 1983;62:997-1003.
6. Jahrling RB, Geisbert TW, Dalgard DW, et al. Preliminary report: isolation of Ebola virus from monkeys imported to USA. Lancet 1990;335:502-5.
7. World Health Organization. Viral haemorrhagic fever in imported monkeys. Wkly Epidemiol Rec 1992;67:142-3.
8. World Health Organization. Ebola haemorrhagic fever in Zaire, 1976. Bull WHO 1978;56:271-93.
9. Sanchez A, Kiley MP, Holloway BP, Auperin DD. Sequence analysis of the Ebola virus genome: organization, genetic elements, and comparison with the genome of Marburg virus. Virus Res 1993;29:215-40.

### Prospects for the Control of Bolivian Hemorrhagic Fever

Bolivian hemorrhagic fever (BHF) was first identified in 1959 as a sporadic hemorrhagic illness in rural areas of Beni department, Bolivia. Clusters of BHF patients were noted the same year, and by 1962 BHF was recognized as a new epidemic infectious disease. In 1963, Machupo virus (a member of the family *Arenaviridae*) was first isolated from patients with acute hemorrhagic fever in San Joaquin, Bolivia (1). Ecologic investigations established the rodent *Calomys callosus*, which is indigenous to the disease-endemic region of northern Bolivia, as the reservoir for Machupo virus (2,3).

Machupo virus infection in *C. callosus* results in asymptomatic infection with shedding of virus in saliva, urine, and feces; 50% of experimentally infected *C. callosus* are chronically viremic and shed virus in their bodily excretions or secretions (2). Although the infectious dose of Machupo virus in humans is unknown, exposed persons may become infected by inhaling virus shed in aerosolized secretions or excretions of infected rodents, by eating food contaminated with rodent excreta, or by direct contact of excreta with abraded skin or oropharyngeal mucous membranes (4). Reports of person-to-person transmission are uncommon; however, hospital contact with a patient resulted in person-to-person spread of Machupo virus to nursing and pathology laboratory staff (5). In 1994, the fatal secondary infection of six family members in Magdalena from a single naturally acquired infection further suggested the potential for person-to-person transmission (Ksiazek et al., manuscript in preparation).

The pathogenesis of BHF, which resembles that of other South American hemorrhagic fevers due to Arenavirus infection (e.g., Argentine hemorrhagic fever), has been described in clinical and pathologic investigations of naturally infected patients (6,7). Experimental infection of rhesus monkeys with Machupo virus demonstrated an incubation period of 7 to 14 days, which is consistent with clinical observations in human infection (8). Early clinical manifestations in humans are characterized by nonspecific signs and symptoms including fever, headache, fatigue, myalgia, and arthralgia. Later in the course of disease (usually within 7 days of onset), patients may develop hemorrhagic signs, including bleeding from the oral and nasal mucosa and from the bronchopulmonary, gastrointestinal, and genitourinary tracts.

During the BHF epidemics in the 1960s, rodent control was recognized as the primary method for the prevention of Machupo virus transmission (9). Since *C. callosus* was frequently found in domestic and peridomestic environments, rodent control measures (e.g., trapping, poisoning) resulted in an immediate reduction in the number of *C. callosus* and control of BHF outbreaks; an epidemic in 1964 ended after 2 weeks of continuous trapping for *C. callosus* in homes of the affected community (10). Rodent control programs became a new priority for health officials in Bolivia, and active interventional programs were carried out for many years by survivors of past BHF epidemics known to be immune to Machupo virus (11).

From 1973 to 1992, no cases of BHF were reported, possibly because of effective control of rodent reservoir populations (12). Since the late 1960s, no epidemics of BHF have occurred that involve rural communities, but recent sporadic cases have been identified in the disease-endemic region (13). Although patients with BHF have been treated at hospitals outside the disease-endemic region, these patients had a history of exposure to Machupo virus in the disease-endemic region or secondary contact with BHF patients who became infected in the endemic region. Additionally, no documented cases of BHF have been exported to other countries.

Concurrently with the lack of identification of BHF patients during the 1970s and 1980s, the emphasis on conducting rodent control programs in the BHF-endemic areas also diminished. Moreover, in recent years, Bolivian health officials have been faced with numerous other public health problems, including diarrheal disease, tuberculosis, Chagas' disease, sexually transmitted diseases, and acquired immunodeficiency syndrome. Thus, local health authorities are confronted with the challenge of allocating limited health resources for the control of BHF as the demand for work with other important diseases increases.

Agricultural activities dominate the economy of northern Bolivia where many workers are employed in farming and animal husbandry (14). Farm workers may reside for prolonged periods in rural areas also inhabited by *C. callosus*, and farm houses constructed with partially open walls may allow rodents access to living areas. Thus, human exposure to infected rodents may occur in and around farm workers' shelters or during work in the fields and grasslands of the BHF-endemic region. Given the projected economic growth in Bolivia, it is likely that agricultural workers' risk for exposure to *C. callosus* will continue and even increase as development modifies the natural habitat of the rodent reservoir leading to increased contact with humans (e.g., focused rodent habitats with increased densities) (15).

Future efforts to control BHF may benefit from recent experience in neighboring Argentina where ongoing work has led to the control of Argentine hemorrhagic fever (AHF), caused by Junin virus, an arenavirus genetically related to Machupo virus. Extensive study of AHF by Maiztegui, Enria, and colleagues has provided new insights into the epidemiology, pathogenesis, treatment, and control of this disease (16,17) and has led to an effective Candid #1 vaccine against Junin virus as well as phase 2 clinical trials that suggest ribavirin may be effective in patients with AHF (18,19). The use of an effective vaccine against AHF and evidence for its cross-protection against Machupo virus suggest that vaccination may play a role in the prevention of BHF for persons at highest risk, such as workers who trap rodents for control programs (20). Intravenous ribavirin has shown promise for the treatment of clinically diagnosed BHF cases subsequently confirmed in the laboratory (Kilgore, manuscript in preparation). Intravenous ribavirin also appeared effective in the treatment of a laboratory-acquired infection with Sabiá virus, a related Arenavirus first isolated in Brazil (21). Ribavirin could be administered to patients whose symptoms meet a clinical case definition with subsequent laboratory confirmation of Machupo virus infection. Local laboratory handling of specimens or testing by effective rapid enzyme-linked immunosorbent assays for antigen and IgM antibodies is ideally performed under biosafety level 4 containment, but use of biological safety cabinets and addition to samples of inexpensive reagents such as Triton X-100, which reduce viral titers, allow the development of capability for real time testing.

The family cluster of BHF patients and later sporadic cases in September and October 1994 highlighted the diagnostic challenge of BHF for clinicians. Even local physicians may rarely evaluate BHF patients, and other diseases (e.g., malaria, dengue fever, and yellow fever) that coexist in the BHF-endemic region may resemble BHF in the early phases of illness. Moreover, no readily available diagnostic tests exist locally to differentiate BHF from other diseases (22). Bolivian health care providers and public health officials recognized the need for education of health care providers and subsequently established a training program aimed at increasing clinicians' recognition of BHF particularly in the disease-endemic region.

The cluster of patients in 1994 also focused public attention on BHF because the illnesses had higher case-fatality rate than other diseases in the region where BHF is endemic. The underrecognition of these illnesses as dangerous and potentially fatal in disease-endemic communities suggests the need for increased public health education to reduce virus exposure and transmission. Proven control measures must be reinforced even in towns affected by large epidemics 30 years ago where younger residents have no recollection of the heavy toll exacted by BHF. Prevention of communitywide epidemics through rodent control programs may be combined with the application of barrier precautions (e.g., gloves, masks) in hospitals or clinics to minimize secondary person-to-person transmission of Machupo virus. After the familial cluster of BHF in 1994, results of rodent trapping confirmed the absence of reinfestation in towns and indicated that the density of rodent reservoirs was not unusually high in areas of probable exposure for the index patient. The absence of communitywide epidemics of BHF suggests that focused rodent control in towns of the disease-endemic region prevented large urban outbreaks. Prevention of sporadic illness in farm workers through widespread elimination of reservoirs may not be feasible, but other measures, such as the administration of Candid #1 AHF vaccine to workers at high risk, may offer a more realistic alternative. Finally, agricultural workers in the disease-endemic region should be taught methods to reduce exposure to rodent reservoirs, especially around rural shelters as a means of reducing their risk of exposure to Machupo virus in the environment.

**Paul E. Kilgore, Clarence J. Peters, James N. Mills, Pierre E. Rollin, Lori Armstrong, Ali S. Khan, and Thomas G. Ksiazek**
National Center for Infectious Diseases, Centers for Disease Control and Prevention, Atlanta, Georgia, USA

### References

1. MacKenzie RB, Beye HK, Valverde L, Garron H. Epidemic hemorrhagic fever in Bolivia: a preliminary report of the epidemiologic and clinical findings in a new epidemic area in South America. J Trop Med Hyg 1964;13:620-5.
2. Johnson KM, MacKenzie RB, Webb PA, Kuns ML. Chronic infection of rodents by Machupo virus. Science 1965;150:1618-9.
3. Johnson KM, Kuns ML, MacKenzie RB, Webb PA, Yunker CE. Isolation of Machupo virus from wild rodent, *Calomys callosus*. Am J Trop Med Hyg 1966;15;103-6.
4. Johnson KM. Epidemiology of Machupo virus infection: III. Significance of virological observations in man and animals. Am J Trop Med Hyg 1965;14:816-8.
5. Peters CJ, Kuehne RW, Mercado RR. Hemorrhagic fever in Cochabamba, Bolivia, 1971. Am J Epidemiol 1974;99:425-33.
6. Stinebaugh BJ, Scholoeder FX, Johnson KM, MacKenzie RB, Entwisle G, DeAlba E. Bolivian hemorrhagic fever: a report of four cases. Am J Med 1966;40:217-30.
7. Child PL, MacKenzie RB, Valverde LR, Johnson KM. Bolivian hemorrhagic fever: a pathologic description. Arch Pathol Lab Med 1967;83:434-45.
8. Kastello MD, Eddy GA, Kuehne RW. A rhesus monkey model for the study of Bolivian hemorrhagic fever. J Infect Dis 1976;133:57-62.
9. Kuns ML. Epidemiology of Machupo virus infection: II. Ecological and control studies of hemorrhagic fever. Am J Trop Med Hyg 1965;14:813-6.
10. Mackenzie RB, Kuns ML, Webb PA. Possibilities for control of hemorrhagic fevers in Latin America. Pan American Health Organization; Scientific Publication No.147:260-265. First International Conference on Vaccines against Viral and Rickettsial Diseases of Man, 1966, Washington, D.C..
11. Mercado R. Rodent control programmes in areas affected by Bolivian hemorrhagic fever. Bull WHO 1975;52:691-6.
12. Pan American Health Organization. Bolivian hemorrhagic fever. Epidemiolog Bull; 1982;3:15-6.
13. Centers for Disease Control and Prevention. Bolivian hemorrhagic fever—El Beni Department, Bolivia. MMWR 1994;43:943-6.
14. United Nations. Statistical yearbook. 39th issue, department for economic and social information and policy analysis, statistical division. New York: United Nations, 1994;201-356.
15. United Nations. Economic and social indicators for latin american countries, including industrialized/agricultural production. Statistical yearbook for Latin American countries and the Caribbean, 1993. New York: United Nations, 1994:238-41.
16. Peters CJ, Johnson KM. Arenaviridae: lymphocytic choriomeningitis virus, lassa virus, and other arenaviruses. In: Mandell GLK, Bennett JE, Dolin R, eds. Principles and practice of infectious diseases, 4th ed. New York: Churchill Livingston, Inc., 1995.
17. Enria D, Garcia Franco S, Ambrosio A, Vallejos D, Levis S, Maiztegui J. Current status of the treatment of Argentine hemorrhagic fever. Med Microbiol Immunol 1986;175:173-6.

18. World Health Organization. Vaccination against Argentine hemorrhagic fever. Wkly Epid Rec 1993;68: 233-4.
19. Enria DA, Maiztegui JU. Antiviral treatment of Argentine hemorrhagic fever. Antiviral Res 1994;23:23-31.
20. Jahrling PB, Trotter RW, Barrero O, et al. Cross-protection against Machupo virus with Candid 1 Junin virus vaccine III. In: Kurstak E, ed. Proceedings of the second international conference on the impact of viral diseases on the development of Latin American countries and the Caribbean Region. Mar del Plata, Argentina, 1988.
21. Barry M, Russi M, Armstrong L, et al. Brief report: occupational exposure to a new arenavirus; Sabiá virus clinical course, treatment and biosafety management. N Engl J Med (in press).
22. Webb PA, Maiztegui JI. Argentine and Bolivian hemorrhagic fevers (South American hemorrhagic fevers). In: Gear JHS, ed. Handbook of viral and rickettsial hemorrhagic fevers. Boca Raton, FL: CRC Press, Inc., 1988.

